# “Every Gene Is Everywhere but the Environment Selects”: Global Geolocalization of Gene Sharing in Environmental Samples through Network Analysis

**DOI:** 10.1093/gbe/evw077

**Published:** 2016-04-29

**Authors:** Marco Fondi, Antti Karkman, Manu V. Tamminen, Emanuele Bosi, Marko Virta, Renato Fani, Eric Alm, James O. McInerney

**Affiliations:** ^1^Laboratory of Microbial and Molecular Evolution, Department of Biology, University of Florence, Italy; ^2^Computational Biology Group, University of Florence, Italy; ^3^Department of Food and Environmental Sciences, University of Helsinki, Finland; ^4^Department of Environmental Systems Science, ETH Zürich, Switzerland; ^5^Department of Aquatic Ecology, Eawag, Switzerland; ^6^Department of Civil and Environmental Engineering, Massachusetts Institute of Technology; ^7^Department of Biology, National University of Ireland Maynooth, County Kildare, Ireland; ^8^Computational Evolutionary Biology, Faculty of Life Sciences, The University of Manchester, United Kingdom

**Keywords:** biogeography, horizontal gene transfer, antibiotic resistance

## Abstract

The spatial distribution of microbes on our planet is famously formulated in the Baas Becking hypothesis as “everything is everywhere but the environment selects.” While this hypothesis does not strictly rule out patterns caused by geographical effects on ecology and historical founder effects, it does propose that the remarkable dispersal potential of microbes leads to distributions generally shaped by environmental factors rather than geographical distance. By constructing sequence similarity networks from uncultured environmental samples, we show that microbial gene pool distributions are not influenced nearly as much by geography as ecology, thus extending the Bass Becking hypothesis from whole organisms to microbial genes. We find that gene pools are shaped by their broad ecological niche (such as sea water, fresh water, host, and airborne). We find that freshwater habitats act as a gene exchange bridge between otherwise disconnected habitats. Finally, certain antibiotic resistance genes deviate from the general trend of habitat specificity by exhibiting a high degree of cross-habitat mobility. The strong cross-habitat mobility of antibiotic resistance genes is a cause for concern and provides a paradigmatic example of the rate by which genes colonize new habitats when new selective forces emerge.

## Introduction

The spatial distribution of microorganisms on the planet is often expressed according to Baas Becking’s famous tenet “everything is everywhere but the environment selects” (Baas Becking 1934). “Everything is everywhere” alludes to the remarkable dispersal potential of microorganisms, whereas “the environment selects” implies that only specifically adapted organisms will thrive and proliferate in a particular environment (Fuhrman 2009). The Baas Becking hypothesis does not rule out the possibility of strong geographic patterns but rather suggests that geography per se does not drive the distribution of species—geographic patterns could simply reflect an association between geography and ecology. Empirical testing of the Baas Becking hypothesis has focused mainly on specific microorganisms and/or specific environments (Reno et al. 2009; Sul et al. 2013). Because most members of microbial communities resist cultivation, understanding of molecular and ecological details of microbial biogeography remains vague (Staley and Konopka 1985; Martiny et al. 2006; Raes et al. 2011; Hanson et al. 2012). However, the recent increase in the number of metagenomes in public repositories offers an opportunity to explore the global distribution of coding sequences, universally shared phylogenetic marker genes, and horizontally transferred genes, including genes of clinical importance such as antibiotic resistance genes (Fondi and Fani 2010).

Furthermore, many studies have highlighted the importance of network theory and approaches based on sequence similarity networks (SSNs) in studying large-scale evolutionary relationships, including the influence of habitat and ecology in the distribution of gene pools, evolution of organisms, and horizontal gene transfer (HGT, Lima-Mendez et al. 2008; Halary et al. 2010; Dagan 2011; Tamminen et al. 2012; Alvarez-Ponce et al. 2013; Forster et al. 2015). However, in most cases, only completely sequenced genomes (including plasmids and phages) were used for these analyses, thus limiting the scope of the studies to mainly cultivable microorganisms or specific phyla (i.e., ciliates). Indeed, often the initial habitat assignment stems from where the organism was first isolated, which may not be its only, or even its preferred, habitat (Hooper et al. 2009).

Here, we empirically test the Baas Becking hypothesis by applying it to genes as well as organisms. By studying 339 metagenomes (pooled into roughly 100 sampling points) using an SSN approach (Fondi and Fani 2010; Halary et al. 2010), we offer a culture-independent view of microbial gene pool commonalities and differences and investigate whether the distributions of genes are limited to particular ecological niches or whether they display a cosmopolitan or geographically defined distribution. Geographical influence on overall patterns of gene distribution is measured as the correlation between the physical distance and the degree of shared homologous sequences between the metagenomes. A positive or negative correlation indicates a distance-effect on global macroscale patterns of gene distributions, whereas absence of such correlation suggests independence between geographical distance and proportion of shared sequences. While gene dispersal may depend on the distribution patterns of microbial species, genes can also rapidly move between phylogenetically distant cells by means of HGT. To test whether the putative horizontally transferred genes follow the distribution of their hosts or form their own distribution, we converted the reconstructed SSN into an HGT network and investigated its main topological features.

By applying a network-oriented analysis pipeline on culture-independent environmental data, we here demonstrate the cosmopolitan distribution of genes and the influence of ecology on their distribution and, in parallel, we show that the same patterns hold for “mobile” genes. Our findings have important implications in several areas of biology, from environmental microbiology to antibiotic resistance, to microbial evolution and to the structure of present day common gene pools.

## Materials and Methods

### Data Set Assembly and Validation

Metagenomic sequences (contigs) used in this work were downloaded from three major repositories, IMG (http://img.jgi.doe.gov/), MG-RAST (http://metagenomics.anl.gov/, Meyer et al. 2008), and CAMERA (http://camera.calit2.net/, Sun et al. 2011). The presence of redundant projects (i.e., the same project deposited in two different repositories) was checked manually and, in those cases, only one of the two projects was maintained. When only sequencing reads were available, shotgun metagenomics assembly was performed. Quality control and removal of identical reads were done with Prinseq (Schmieder and Edwards 2011). For most of the samples, assembled contigs were available on the public repositories mentioned above. In those cases where (Roche 454) shotgun DNA sequences were available, assembly was carried out using Phrap using the default parameters (Machado et al. 2011).

A total of 339 metagenome projects (supplementary material S1, Supplementary Material online) were retrieved, processed, and analyzed. Each of the retrieved projects was associated with a habitat, according to its sampling point as indicated in the metafiles associated with each of the project. Nine main categories were defined for sampling habitats, including soil, seawater, inland-water, wastewater, host, air, bioremediation, biotransformation, and sludge waste. Samples for which a clear habitat of the corresponding sampling point was not available were labeled as “Unknown.”

Additionally, for each metagenome the exact sampling point (latitude and longitude) was retrieved (Global Positioning System [GPS] coordinates). The physical distance (*d*, expressed in km) among the different sampling points was computed from their GPS coordinates using the spherical law of cosines, that is:

d= acos (sin(ϕ1)*sin(ϕ2) + cos(ϕ1)*cos(ϕ2)*cos(Δλ)) *R,

where ϕ1 and ϕ2 represent latitude values (in degree) of points 1 and 2, Δλ represents the difference between longitude values of points 1 and 2, and *R* is the earth’s radius (mean radius = 6,371 km). In cases in which we found different metagenome projects (i.e., different naming and different number of sequences but same habitat) with (almost) identical sampling points (i.e., within a radius of 20 km), the corresponding projects were pooled into a single sequence fasta file. Ribosomal sequences were removed from each sequence data set using Ribopicker software (Schmieder et al. 2012) with default parameters.

At the end of the data set assembly and checking procedures, 97 Fasta files were obtained, embedding a total of 1,019,781 contig sequences (longer than 1,500 bp). These were used as input for homology-based network construction pipeline. Fasta files and scripts used in this work have been made publicly available at http://sourceforge.net/projects/metanetwork/.

### BLAST Searches and Evolutionary Distances Computation

Homology searches among sampled contigs were performed using BLASTp and BLASTn from the BLAST suite (Altschul et al. 1997). Only hits longer than 500 bp and with an *E* value lower than 1e ^−^^100^ were considered for further analysis (multiple hits among two contigs were counted only once and no constraints on the alignment coverage were imposed). Furthermore, several identity thresholds were considered, that is, 70%, 80%, 90%, 95%, and 99%. A summary of the main features of contigs embedded in our data set and BLAST hits is reported in supplementary fig. S1, Supplementary Material online.

BLAST outputs were then postprocessed in the form of undirected networks (accounting for the different identity thresholds). Two different kinds of network were obtained 1) metagenomic network and 2) contig network. In the first type of network, nodes represent single metagenome projects (or metagenome project pools), whereas links represent the amount of BLAST hits they share. In the second kind of network (“contig network”), every node represents a contig and two nodes are connected if a significant hit was retrieved among them.

Five different identity thresholds were selected (70%, 80%, 90%, 95%, and 99%) and an alignment length threshold of 500 bp was set to place links between two different metagenomes (nodes). It must be noted that the size of the different metagenomes in the data set may influence their degree (i.e., the number of their connections) in the network; indeed, larger metagenomes might have higher probability to be more connected in the graph, just by random chance. To overcome this issue, we also computed a normalized value for each link, dividing the actual number of BLAST hits by the sum of the number of sequences possessed by the two metagenomes and evaluated the correlation between connectivity and number of sequences for each metagenome in the normalized network. A Pearson product moment calculation over the original (not normalized graph) revealed a (low) positive correlation among connectivity and sample size (Pearson-product-moment correlation = 0.126, *P* value < 2.2e-16). The same calculation repeated after normalizing link values produced a Pearson-product-moment correlation of 0.044, with a *P* value of 0.002117, suggesting a minor size effect on the computed similarity network. All BLAST postprocessing was performed with in-house-developed Perl and Python scripts.

To account for the actual amount of sequence possessed by each sample (and not only the number of contigs possessed), we performed an alternative normalization process, dividing the number of BLAST hits between two nodes by the number of bases (not the contigs) possessed by the two corresponding samples. General trends computed in the rest of the article were not affected by the normalization procedure implemented since the clustering of the different samples was still influenced by ecology rather than by their physical distance.

To test whether a correlation exists among the number of BLAST hits shared by two metagenomes and their geographical distance, the Pearson-product-moment correlation was calculated. Results obtained (Pearson-product-moment correlation = −0.038, *P* value = 06 × 10 ^−^^3^) revealed the absence of a statistically significant correlation among physical distance (expressed in km) and the number of shared hits (supplementary fig. S2, Supplementary Material online).

To account for the evolutionary distances among the (coding) sequences in our data set, we have also implemented the following pipeline. First, we have performed an all versus all BLAST of the coding sequences embedded in our metagenomes data set. Next, we extracted 100 000 groups of homologs among the different samples using an *E*-value threshold of 1e-70. At this stage, to avoid considering underrepresented samples, we focused our analysis only on the most represented samples (i.e., inland water, host associated, sea water, and soil). Such a low *E*-value threshold was used to retrieve highly similar sequence from the different data sets that could facilitate accurate sequence alignment and distance calculation in the next steps of the pipeline. Identified groups of orthologs were then aligned using Muscle (Edgar 2004a, 2004b) and the resulting multialignments were automatically edited using Gblocks (Talavera and Castresana 2007) to remove poorly aligned regions. Edited multialignments were then used as input for the distmat tool of EMBOSS (Rice et al. 2000) suite, leading to the creation of one distance matrix (according to Jukes–Cantor model [Jukes and Cantor 1969]) for each group of homologs shared by the samples. From these we calculated and compared the evolutionary distances among genes shared by the same samples and among those shared by samples from different niches.

### Identification of Marker Genes

Universal phylogenetic marker genes were identified from the metagenomes using the fetchMG program version 1.0 (Sunagawa et al. 2013). All identified marker genes from one metagenome were pooled and used in network analysis. Connections between metagenomes were normalized with the sum of sequences in the two metagenomes, as described previously. To test whether a correlation existed among the number of shared marker genes by two metagenomes and their geographical distance, the Pearson-product-moment correlation was calculated (supplementary fig. S3, Supplementary Material online).

### Network Analysis and Visualization

Graph topology and statistical tests were performed with the igraph (v. 1.0.0) library of the R statistical package (v. 3.1.3, http://www.r-project.org/) and in-house-developed Perl and R scripts. The main graph metrics evaluated in this work were betweenness centrality, clustering coefficient, closeness centrality, and assortativity. Briefly, betweenness is a centrality measure that indicates which nodes are holding the network together; nodes with high betweenness values can be bridges between otherwise disconnected regions of the network. The clustering coefficient measures the extent to which the neighbors of a given node are interlinked. We used this coefficient as an indicator of cohesiveness around a node neighborhood. The closeness of a node is the inverse of its average distance to all other nodes in the graph. The higher the closeness, the more central is the node. Finally, assortativity measures the tendency of nodes with the same label (the source ecological niche in our case) to preferentially connect with one another in the graph (Newman 2003). If a network has perfect assortativity (*r* = 1), then all nodes connect only with nodes of the same kind. If the network has no assortativity (*r* = 0), then any node can randomly connect to any other node. If a network is perfectly disassortative (*r* = −1), all nodes will have to connect to nodes with different degrees.

Statistical support to these centrality measures was provided through randomization of the original graph. More in detail, here the null model reflects the possibility that interactions are equally likely between any pair of nodes in the graph. In other words, our stochastic null model has no centrality structure. In this case, our randomized networks contained the same nodes, but edges were rearranged randomly among them (edges rearrangement). Statistical tests (e.g., Mann–Whitney test) were carried out each time to infer whether original and randomized networks differed significantly./

Network visualization and postprocessing were done using the Cytoscape and Gephi software (Bastian et al. 2009; Kohl et al. 2011). The GeoLayout Gephi plugin was used to build geocoded graphs of gene sharing.

### Computational Strategy for Clusters Identification and Testing

To identify network clusters in the metagenomes network, a community detection algorithm (MCL, van Dongen and Abreu-Goodger 2012) was first applied to the graph. The main parameter of this algorithm is the inflation factor (IF) that modulates cluster granularity. To choose the optimal IF (i.e., to select the proper trade-off between clusters size and their overall homogeneity), we explored values ranging from 1.2 to 5 by steps of 0.2 and estimated cluster homogeneity by computing the average intracluster cluster coefficient (ICCC) at every step. Briefly, the clustering coefficient measures the “cliquishness” around a node; hence, its average over the nodes of a cluster can be used as a measure of the cluster homogeneity. ICCC is computed considering only the edges within clusters and, in principle, a clustering result that maximizes the ICCC produces more homogeneous graphs. Supplementary fig. S5, Supplementary Material online, shows the trend of the ICCC and the number of clusters at different IF values for the 90% network. As expected, the number of clusters increases as the IF increases, whereas the opposite holds for ICCC. The peak at inflation value of 1.4 suggests that this clustering solution is the best trade-off between network fragmentation and cluster size (supplementary fig. S4, Supplementary Material online). Additionally, this threshold was shown to perform reasonably well also for the networks obtained at different network clustering, allowing the identification of eight major clusters (i.e., with at least two nodes).

To test the presence of a correlation between the clustering of the different nodes (metagenomes) and their source habitat, we implemented a computational strategy similar to the one applied by Lima-Mendez et al. (2008). Once the clusters were identified in the network, we evaluated the correspondence between such clusters and the source habitat of the different nodes represented by the different nodes. In other words, we evaluated whether metagenomes belonging to the same ecological niche tended to cluster together or not in a significant manner.

Three different measures are classically adopted to evaluate the overlap between some kind of classifications (in our case network clustering and source ecological niche): recall (R), precision (P), and accuracy (A). R evaluates whether all nodes of a given habitat are found in the same cluster (*R* = 1) or there are found embedded in different clusters of the network (*R* < 1). Conversely, P measures how well a given cluster corresponds to its best-matching habitat; a value of 1 indicates that all nodes in the cluster belong to the same habitat. Similar to Lima-Mendez et al. (2008) from the class- and cluster-wise statistics, the clustering-wise statistics were computed as the weighted means over all habitat/clusters of the class/cluster-wise values. The geometric mean of R and P gives the accuracy measure. Results obtained with this approach were compared to random expectations performing 1,000 permutation tests by shuffling labels of the nodes in the network while maintaining the structure of the network. The null hypothesis underlying this approach is that any node (group of sequences) can occupy any network position (i.e., could cluster with any other node in the network). Accordingly, during our randomizations, the network structure is held constant and the node labels are permuted. A graph sampled with this approach retains all network traits of the empirical graph and this enables assessment of whether the node characteristics depend on the structure of the graph. For each of the permutations, the same statistics (R, P, A) were computed and finally compared to the observed ones.

### Contig Taxonomic Annotation and Source Molecule Identification

Each contig of the metagenome data set was assigned to the (putative) corresponding genus using the approach implemented in RAIphy (Nalbantoglu et al. 2011).

Finally, since RAIphy is a semisupervised method that relies on reference genomes, sample types that have better representative set of sequenced genomes may achieve higher supervised classification rates and will tend to connect with each other more frequently. To avoid possible biases due to the use of a semisupervised method, we also implemented a composition-based method (using tetranucleotide frequency distributions) for the identification of (putative) HGTs. Briefly, for each match between two contigs, the tetranucleotide frequencies of the flanking regions were compared as described in Teeling et al. (2004). Only matches where the flanking region was at least 1,000 bp and the Pearson correlation coefficient between the tetranucleotide profiles was below 0.7 were considered as putative HGT events.

The most likely source molecule of each contig (i.e., plasmid or chromosome) was identified using the composition-oriented software cBar (Zhou and Xu 2010). Both tools were used with default parameters.

### ORF Identification and Functional Annotation

ORFs were identified using the FragGeneScan software (Rho et al. 2010). Functional annotation of identified ORFs was performed using hmmscan from HMMER (version 3.1b2 [Finn et al. 2011]) with an *E*-value cut-off of 0.1 and probing the Pfam database (Finn et al. 2014). Antibiotic Resistance (AR)-related genes were identified through BLAST (blastp) searches against Antibiotic Resistance Database (Liu and Pop 2009).

### Adjacency Matrix Construction

The adjacency matrix accounting for the degree of interconnections among samples from the different environments was computed as follows:

For each habitat, the proportion of connections of that habitat with all the other habitats has been computed. The proportion of connections connecting habitat A with habitat B (${PC}_{A,B}$) is given by this formula:

$${\text{PC}}_{A,B}=\frac{\text{Weight}({\text{Edge}}_{A,B})}{\sum_{i}\text{Weight}({\text{Edge}}_{A,i})}$$

The PC index ranges from 0 to 1 and measures the specificity of the connection between one habitat in respect to the others. Since the denominator represents the amount of sequences in one of the two analyzed samples, this measure is specific to each of the analyzed environments and is not symmetric ${PC}_{A,B}\neq {PC}_{B,A}$. The PC values have been organized in the form of a matrix where all these values have been normalized by computing the row *Z* score, which means that rows of the matrix are centered and scaled by subtracting the mean of the row from every value and then dividing the resulting values by the standard deviation of the row.

$$Z_{i}^{\text{row}}=\frac{X_{i}-{µ}_{\text{row}}}{\sigma_{\text{row}}}$$

## Results and Discussion

### General Features

**Fig. 1.—:**
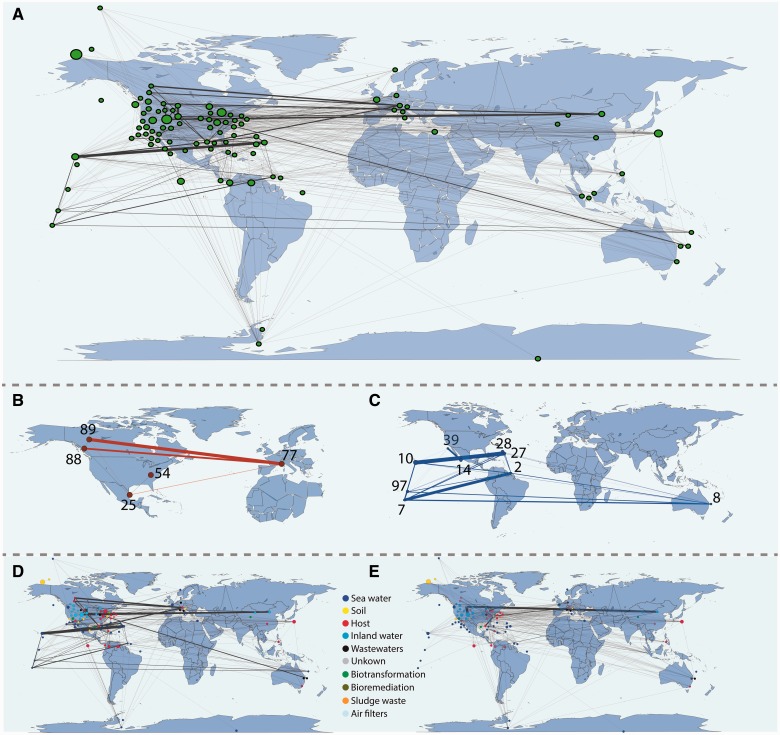
(*A*) Overall SSN among the 97 sampling points together with their geographical positions. Each node represents a metagenome project and the links represent the presence of homologous sequences between them. Node and link sizes are proportional to the number of sequences embedded in the sample and the (normalized) number of shared sequences, respectively. In (*B*) and (*C*) specific study cases are reported (see text for details) for host-(red nodes) and sea-water (blue)-derived samples. The connections among samples from the same ecological niche and those among samples from different ecological niches are shown in (*D*) and (*E*), respectively.

We built an SSN using metagenome sequences from 97 sampling sites (representing 339 metagenomic projects, see supplementary material S1, Supplementary Material online) where nodes represent sampling points and links reflect the number of shared homologous sequences (see Materials and Methods for network construction details). We used different sequence identity thresholds in building these SSNs (i.e., 70%, 80%, 90%, 95%, and 99%). Results presented here refer to the 90% network, although the results are valid for all identity thresholds (see supplementary material S1, Supplementary Material online). In figure 1, the extent of sequence sharing among the different samples is presented as a network, together with the geographical location of each sampling site. To test whether physical distance and the number of homologous DNA fragments shared by the different metagenomes correlate, we calculated Pearson-product-moment correlation coefficients for samples from different (Pearson Correlation Coefficient [PCC] = −0.038 and *P* value = 6 × 10 ^−^^3^) and same habitats (from PCC = −0.2 in soil samples to 0.04 in fresh water samples, *P* values < 6 × 10 ^−^^3^; supplementary fig. S2, Supplementary Material online). Therefore, physical distance at the spatial resolution provided by the available metagenomes does not explain the distribution of the links in the metagenome-derived SSN, suggesting a relatively marginal role of physical distance in the shaping of the biological relationships. Exemplars of this situation are reported in figure 1*b* and *c* for host- and sea water-derived samples. Metagenomes of the subnetwork of figure 1*b* (samples no. 77, 25, 88 and 89, see supplementary material S2, Supplementary Material online), although connected to almost all the other metagenomes in the network, share many more sequences among themselves. The sequences embedded in these metagenomes were obtained from microbiomes of geographically distant Arthropods: *Dendroctonus ponderosae* (samples 88 and 89), *D. frontalis* (sample 25), *Xyleborus affinis* (sample 77), and *Sirex noctilio* (sample 54). We observed a similar trend in geographically disparate specimens of sea squirt *Ciona intestinalis* (Dishaw et al. 2014), consistent with the selection of a core community by that particular ecosystem. We observed the same feature for metagenomes displayed in figure 1*c* (samples no. 2, 97, 10, 39, 14, 28, 27, 2, and 8, see supplementary material S2, Supplementary Material online), all from seawater samples and all sharing heavy connections despite most being separated by large geographical distances. Accordingly, we speculate that the similarity of the ecological niches in which samples were collected explains the high level of gene sharing among these two sets of metagenomes. Figure 1*c* also shows that, within samples sharing the same source niche, some nodes that are close in the network (e.g., 10, 7, and 97) display fewer connections among them in respect, for example, to those shared with nodes 28 and 2 (being far away in the map). This, in turn, might suggest the limit of using physical distances as a proxy for estimating the “real” distance among gene pools. Indeed, other barriers and forces (besides geographical distance) might account for the actual dispersal. This is the case, for example, of sea currents that may contribute to creating quite different environments in two close points in the network of metagenomic samples. Similarly, mountains might create a separation among physically close terrestrial DNA pools. On the other hand, these features are quite hard to be confidently modeled on a large, global scale as the one used in this work.

**Fig. 2.—:**
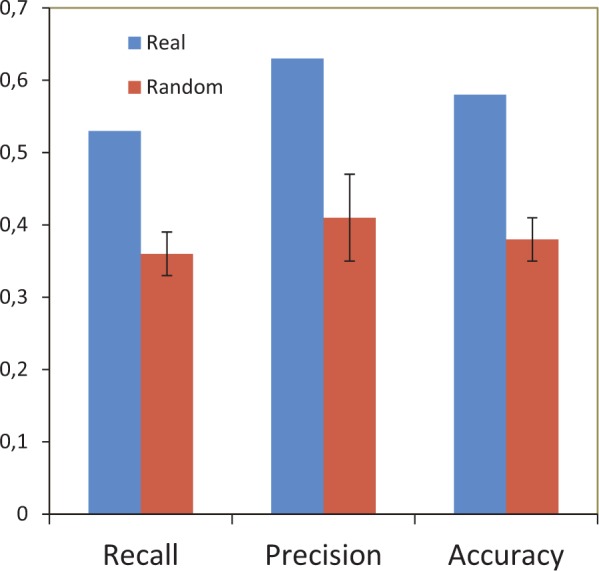
Recall, precision, and accuracy values for real and random network at 90% sequence identity threshold.

A preliminary visual inspection of the network revealed that samples from same ecological niches (fig. 1*D*) are more tightly connected than samples from different niches (fig. 1*E*). Thus, to explicitly test the ecological niche versus geographical distribution hypotheses, we evaluated the correlation between the grouping of the different metagenomes (i.e., the habitat composition of the major clusters in the network of fig. 1) and their source habitat. We first clustered the metagenomes according to the Markov Cluster (MCL) algorithm (see Materials and Methods) and then evaluated whether metagenomes belonging to the same ecological niche tended to (significantly) cluster together using recall (R), precision (P), and accuracy (A) measures. This analysis (fig. 2) revealed relatively high values of both R and P across all the different networks (average *R* = 0.588 and average *P* = 0.71). A similar trend was observed also when measuring clustering accuracy (A) (fig. 2). Such high values of P, R, and A were never obtained during 1,000 random permutations (label shuffling, see Materials and Methods) of the original networks, giving a *P* value estimate < 10 ^−^^3^. The same results were observed for networks obtained with lower sequence identity thresholds (supplementary fig. S6, Supplementary Material online) and when evolutionary distances were considered for a set of 10,000 randomly sampled coding sequences in the data set (supplementary fig. S7, Supplementary Material online).

**Fig. 3.—:**
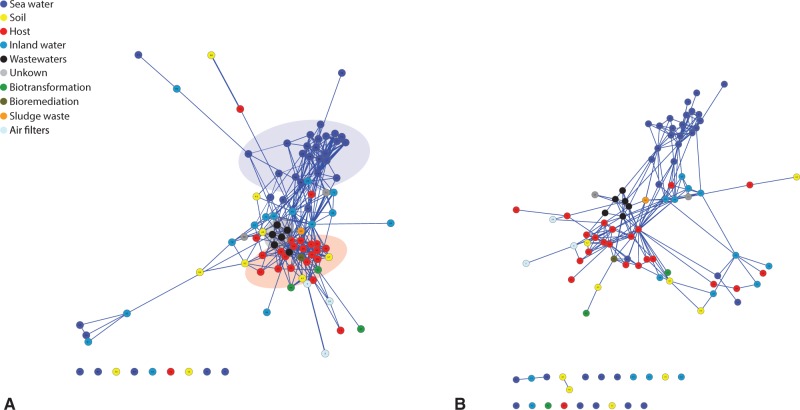
(*A*) Force-directed layout representation of the metagenome network (at 90% sequence identity threshold). Each metagenome is colored according to its source habitat as indicated in the legend and major coherent clusters are highlighted. (*B*) The putative HGT network derived from network shown in (*A*) (see text for details on HGT network construction).

Additionally, assortativity was used to evaluate the tendency (if any) of nodes of the same type (i.e., sequences from the same source habitats) to cluster together in the network. Briefly, assortativity coefficient measures the preference for a network's nodes to attach to others that share a particular attribute (source environment in our case) and can be comprised between −1 (disassortative network) and 1 (assortative network). Assortativity for the network in figure 3 was found to be 0.157, thus confirming a general pattern of preferential connections between nodes of a particular ecological niche. Importantly, higher assortativity values were never encountered when (1,000) randomization of the original network were performed (edge rearrangement, see Network Analysis and Visualization), allowing to infer a rough estimation of a *P* value lower than 10 ^−^^3^.

From this we conclude that the source habitat of the different sequence samples is a key factor in determining their clustering within the different SSNs.

A force-directed layout of the network (fig. 3*a*) reveals a clear separation between sea samples (in dark blue) and samples coming from other sources such as host (red), soil (yellow), waste waters (black), and air filters (light blue). Interestingly, inland-water samples (blue) appear to lay half way between these two major clusters. As listed in table 1, metagenomes from inland water samples possess the highest betweenness values in the SSN in comparison to all the other sample sources, expressing that these nodes have a central position in the network and that, in turn, they serve as connectors among otherwise separated regions of the network (Mann–Whitney *U* test, *P* values in table 1). These results were confirmed by randomizations (edge replacement, see Network Analysis and Visualization) of the original graph (table 1) according to which inland water metagenomes, and (to a lower extent) sea water metagenomes, have betweenness centrality values higher than is expected by chance. Inland water metagenomes are also less prone to form clusters within the network, since they show, on average, the lowest clustering coefficient (Mann–Whitney *U* test, table 1). Inland water metagenomes possess also the highest closeness centrality values in the SSN (Mann–Whitney *U* test, table 1). This suggests that, in water, bacteria from different origins (human, animal, and environmental) may be able to mix, co-exist, and travel to an extent that is higher than in other ecological niches. This could give rise to exchange and shuffling of genes, genetic platforms, and genetic vectors (Baquero et al. 2008). This result confirms and extends previous findings on the horizontal flow of the plasmid encoded resistome (Fondi and Fani 2010).

**Table 1:** Centrality Measures in Relation to Sample Environmental Origin in Observed and Random Networks

Network Metric	Soil		Sea		Host		Inland water	
	Real	Random	Real	Random	Real	Random	Real	Random
Betweenness	4.6	16.8(6.02)	52.46	42.20(4.05)	50.52	61.14(5.24)	102.67	63.76(6.9)
	*P* = 2*10^−3^		*P* = 2*10^−2^		*P* = 2*10^−2^			
Closeness	0.42	0.44(0.008)	0.46	0.48(0.07)	0.49	0.50(0.009)	0.50	0.48(0.01)
	*P* = 1*10^−3^		*P* = 9*10^−3^		*P* = 4*10^−3^			
Clustering c.	0.68	0.18(0.03)	0.6	0.24(0.03)	0.56	0.24(0.02)	0.39	0.20(0.03)
	*P* = 1*10^−3^		*P* = 3*10^−1^		*P* = 2*10^−3^			

Note.—Values in parentheses after randomized values indicate standard deviation. Values after real values for soil, host, and sea metagenomes indicate *P* values for comparisons to inland water samples (Mann–Whitney *U* test).

As shown in figure 3*a*, nine metagenomes remained disconnected from the overall network. These metagenomes included five seawater samples, two soil samples, one host, and one inland water samples. Not surprisingly, these metagenomes embed fewer sequences than others present in the data set. Indeed, although it has been shown that the metagenome size has a negligible effect on the overall connectivity within the network (see supplementary material S1, Supplementary Material online, and Materials and Methods), some exceptions may still exist. These metagenomes are connected to the others at lower identity thresholds (data not shown).

### HGT Networks

The extent of sequence sharing among the metagenomes can be partially explained by the overlapping taxonomical space of the different samples; indeed, similar habitats may tend to be colonized by the same major taxonomical groups. This latter observation is supported by the results obtained repeating the same analysis pipeline for marker genes retrieved in the studied metagenomic samples (supplementary fig S4, Supplementary Material online) and likely with a reduced susceptibility to HGT. Nevertheless, the assembled data set permits us the opportunity to assess the relationships (if any) between physical proximity, ecological niche, and HGT. To account for this task, a second set of networks was constructed, accounting for putative HGT events among the analyzed sequence data sets. We identified putative HGTs as blocks of nearly identical DNA (≥500 nucleotides and ≥98% sequence identity) in otherwise distantly related contigs (i.e., contigs from different genera inferred by a composition-based, semisupervised, taxonomic binning algorithm). Since the method adopted for taxonomic binning of metagenome sequences is mainly suited to microbial sequences (Nalbantoglu et al. 2011), only prokaryote to prokaryote putative gene exchanges will be considered in the following sections. Importantly, trends in sequence sharing described below were observed also when a composition-oriented method (based on the evaluation of differences in tetranucleotide frequency distribution between two contigs, see Contig taxonomic annotation and source molecule identification) was used for the identification of (putative) HGT.

The network of HGT among metagenomes is reported in figure 3*b*, displaying a topology very similar to the network of gene sharing (fig. 3*a*) although, as might be expected, possessing fewer links. The HGT network also proves that sequence sharing between metagenomes is not just due to overlapping taxonomical space. To further investigate the HGT network, we built a second type of network in which each node represents a single contig, whereas links account for (putative) HGT events. This network contains 34,555 nodes (contigs) and 34,398 edges (putative HGT events, supplementary material S3, Supplementary Material online) and can be divided into 8,017 connected components (CC), the great majority embedding only few contigs (≤10). We identified 46 larger CCs, embedding 50 or more contigs. Functional annotation was missing for 38% of the genes involved in putative HGT events. Among those that were successfully annotated using Pfam database, the two most represented functional categories were ABC transporters and transposase DDE domain. Considering the biological role of genes embedded into these categories (resistance to xenobiotics and horizontal transfer of genes) this finding highlights the dangerous implications of the horizontal flow of genes in the spreading of microbial resistance (and resistance to xenobionts in general) in natural environments (Baquero et al. 2008; Fondi and Fani 2010). Two examples of this are provided below.

**Fig. 4.—:**
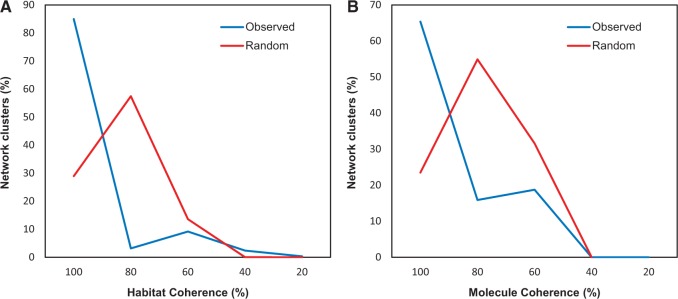
Composition of network clusters in terms of habitat and molecule categories. One hundred percentage values on the *X* axis indicate clusters with contigs belonging to the same category; conversely, lower values indicate more heterogeneous clusters (i.e., contigs belonging to different habitat or to different molecules). The cluster composition is shown for (*A*) habitat coherence and (*B*) molecule coherence (i.e., plasmid–plasmid and chromosome–chromosome).

**Fig. 5.—:**
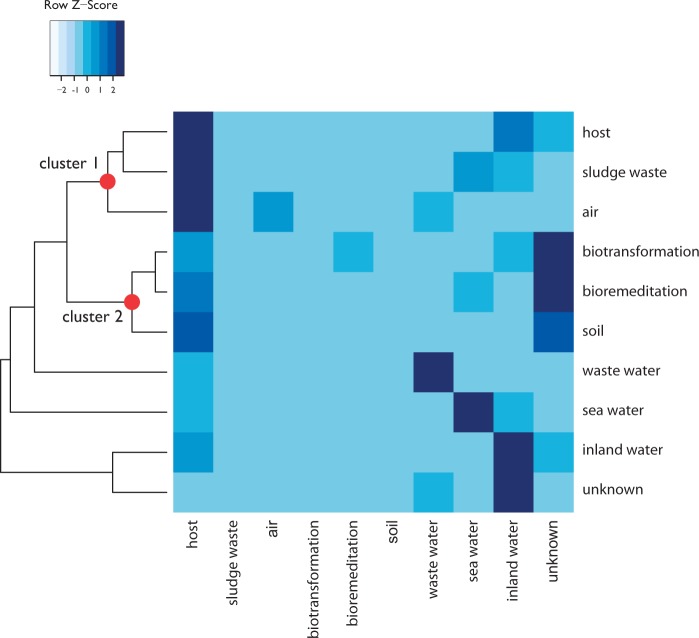
Adjacency matrix showing the relationships among the different habitat types in the putative HGT events network. For each habitat, the proportion of connections of that habitat with all the other habitats has been computed. The proportion of connections connecting habitat A with habitat B (${\text{PC}}_{A,B}$) is given by this formula:${\text{PC}}_{A,B}=\frac{\text{Weight}({\text{Edge}}_{A,B})}{\sum_{i}\text{Weight}({\text{Edge}}_{\text{A},\text{i}})}$Since the denominator represents the amount of sequences in one of the two analyzed samples, this measure is specific to each of the analyzed environments and is not symmetric (${\text{PC}}_{A,B}\neq {\text{PC}}_{B,A}$). Color gradient within the matrix refers to the proportion of connections of contigs from a given habitat with all the others from other habitats, with lighter tones representing less abundant interconnections among the corresponding habitats.

To investigate the influence of ecology shaping the HGT network, we estimated whether each CC was either homogeneous or heterogeneous in terms of the habitat of the embedded contigs. Results shown in figure 4*a* revealed that almost 90% of the CCs (6,814 CCs) contain contigs belonging to the same environment. Heterogeneous clusters are less frequent, although interesting exceptions do exist (see below). The observed distribution of homogeneous clusters was compared against the (averaged) distribution of the same measure from 1,000 networks, obtained through random label reshuffling (see Computational strategy for clusters identification and testing). The distinctness of the two distributions is shown in figure 4*a* and was assessed by a Mann–Whitney *U* test (*P* value < 2.2e-16). A high number of interconnections inside each of the examined habitats (e.g., host–host and sea water–sea water) were observed for most of the samples (fig. 5; see below), in agreement with overall samples clustering reported in figure 4*a* and with previous findings concerning the possible presence of barriers or trends to HGT (Popa and Dagan 2011). According to this whole body of data, ecology seems to exert a broad influence on recent gene exchange in environmental samples. This is in agreement with the theory according to which ecological similarity shapes networks of gene exchange by selecting for the transfer and proliferation of adaptive traits or by increasing physical interactions between community members (Aravind et al. 1998; Caro-Quintero et al. 2011; Smillie et al. 2011). For example, strong geographical differentiation apparently caused by recent gene transfer among co-occurring bacteria was observed for *Vibrio* representatives (Boucher et al. 2011).

An adjacency matrix was built to explore more thoroughly the interconnections that link sequences from different habitats and common patterns of gene exchange among samples retrieved from different ecosystems (fig. 5). Two major clusters can be identified on the basis of the dendrogram topology (Clusters 1 and 2 in fig. 5). Contigs embedded in each of these clusters have similar connections toward the other environments present in the HGT network. This suggests the presence of a common pool of genes in ecosystems embedded in these clusters. Cluster 1, for example, embeds Host, Sludge waste, and Air ecosystems. This particular clustering is supported by Smillie et al. (2011) and studies showing that fecal coliforms and other animal pathogens are indeed present in sludge waste samples (Jones 1980; De Luca et al. 1998; Shanahan et al. 2010) and that opportunistic pathogens commonly isolated from human-inhabited environments have been identified in airborne environments (Tringe et al. 2008). Also, the fact that activated sludge microbiomes are characterized by high microbial density and high levels of various HGT associated traits (e.g., AR-related genes and plasmids/integrons/transposons) (Schluter et al. 2007; Zhang et al. 2011) indirectly supports the observed clustering of sludge waste samples together with microbes from other (diverse) ecological niches (e.g., clinical environment). Similarly, Cluster 2 contains ecosystems that embed overlapping microbial communities (i.e., biotransformation, bioremediation, and soil environments) and thus showing similar patterns of interconnections against microbes from other ecosystems.

**Fig. 6.—:**
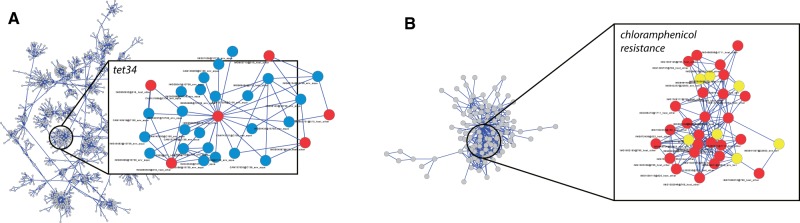
Examples of putative cross-habitat HGT events among contigs (nodes) embedding (*A*) tetracycline resistance determinants and retrieved from inland waters (blue nodes) and host (red nodes) and (*B*) chloramphenicol resistance in host (red) and soil (yellow) derived samples.

Exceptions to ecologically homogeneous clusters can be highlighted within our data set. Two paradigmatic examples of cross-habitat putative HGT were chosen in the overall putative HGT network and are shown in figure 6. In detail, figure 6a reports putative HGTs among contigs embedding tetracycline resistance determinants (*tet*34) in samples isolated from host and inland waters. Tetracycline resistance is often associated with conjugative transposons or other transferable elements (e.g., pheromone-inducible plasmids) (Clewell et al. 1995; Dunny et al. 1995) and plasmid-mediated HGT events involving such determinants have been previously identified (Fondi and Fani 2010; Bosi et al. 2011). Similarly (fig. 6*b*), contigs embedding chloramphenicol resistance determinants belong to samples of very different origin (soil and host). This latter finding shows possible pathways for cross-habitat chloramphenicol-resistance propagation in the environment and is in line with previous observations on swine feedlot wastewater as a possible source of chloramphenicol-resistance genes (Li et al. 2013) and the overall capability of this class of genes to undergo HGT (Sermonti et al. 1978; Takamatsu et al. 2003). Taken together, these two cases show that interhabitat barriers and taxonomic distance can be overcome by certain genes since phylogenetically unrelated bacteria, and those inhabiting distinct environments were found to share common antibiotic resistance determinants, probably as a result of (one or multiple) HGT event(s) (Halary et al. 2010; Smillie et al. 2011).

The network-based approach adopted here allows testing the role of plasmids and chromosomes in the overall gene exchange pattern within environmental samples. Indeed, the importance of plasmids and chromosomes in shaping the microbial HGT network has been assessed in recent works (Halary et al. 2010; Smillie et al. 2011). Halary et al. (2010) showed that gene sharing mostly occurs among molecules of the same type (molecule coherence), meaning that plasmid-plasmid and chromosome–chromosome gene sharing is more frequent than cross-molecule sharing. Accordingly, we investigated whether contigs embedded in the same CC belonged to the same or different molecules (i.e., plasmids or chromosomes). Contig sequences were assigned to their source molecule adopting a composition-based strategy as implemented in cBar (Zhou and Xu 2010) and the source molecule composition of each cluster was evaluated. Results reported in figure 4b show an overall coherence within the CCs identified in the network. In particular, 5,199 CCs (∼65% of all the CCs) are highly homogeneous: more than 90% of the embedded contigs belong to the same type of DNA molecule. Conversely, heterogeneous clusters (those in which contigs are almost evenly distributed among the two types of molecules) represent 24.3% of the total number of clusters. Again, the observed distribution of homogeneous clusters was compared against the same (averaged) distribution obtained from 1,000 networks, obtained through label reshuffling (red line in fig. 4*b*). The distinctness of the two distributions was assessed by a Mann–Whitney *U* test (*P* value < 2.2e-16). This finding indicates that DNA pools are mainly transferred between molecules of the same type.

Notably, general trends (i.e., molecule and habitat coherence) among the various clusters were not affected by the method used for estimating the number of HGT events as adopting a composition-based (i.e., tetranucleotide frequencies, see Materials and Methods) approach led to the same overall results (data not shown).

## Conclusions

By adopting a similarity network approach on a comprehensive set of environmental sequences, we revealed the absence of an overall distance effect in the level of sequence sharing among microbial samples; even distant microbial communities may share more homologous sequences than geographically closer DNA pools. Metagenome gene composition is therefore strongly affected by ecology. Interestingly, inland water samples occupy a “bridge-like” position in the overall metagenome network (fig. 3*a*). Hence, despite maintaining their own (specific) gene pool as assessed by clustering analyses, these samples connect microbial communities that otherwise would remain disconnected (e.g., host and seawater samples). This is in agreement with previous findings on the horizontal flow of plasmid genes (Fondi and Fani 2010) and speculations on the role of aquatic environments in the spreading of AR-related determinants (Baquero et al. 2008). These trends were confirmed when the SSN was converted into a putative HGT network by maintaining only those connections linking very similar sequences (identity ≥ 98%) in distantly related microorganisms (i.e., belonging to different genera). Ecology strongly influences the network of HGT in microbes even when samples not strictly related to human are considered, as has also been preliminarily observed in terrestrial and aquatic environments (Hooper et al. 2008). Moreover, HGT events mainly involve molecules of the same kind (i.e., either plasmids or chromosomes) with promiscuous gene exchange being less frequent.

Our work shows the possible use of SSN for studying patterns in microbial ecology and also lays foundations for integrating such networks with other environmental parameters (e.g., temperature, pH, pressure, and physical barriers) on the structure of the gene sharing and HGT networks. Finally, our findings provide support for the Baas Becking hypothesis (formulated in 1934), suggesting that it also applies to genes, besides microbes for which it was originally formulated. Overlapping microbial gene pools are likely to be found in widely geographically disparate environments, and tighter associations are observed among gene pools from similar habitats. This holds true regardless of microbial evolutionary lineages (i.e., their common evolutionary history) since we have shown that the same patterns of common gene pools still remain when only genes likely shared by means of HGT events are maintained in the network. This suggests that it is not so important which organism transcribes and translates a gene and it matters more where that organism is located, demonstrating that at least some genes act as public goods (McInerney et al. 2011). Accordingly, they are available for all organisms to integrate into their genomes although the kind of ecological niches occupied and the type of informative molecules harboring them might impose some constraints on the overall possibility of gene pools to undergo HGT. Finally, besides drafting an overall scheme of pathways for the global distribution of gene pools, results presented here provide important biological insights into the spreading of antibiotic-resistance-related genes across multiple hosts and habitats.

## Supplementary Material

Supplementary Data
