# Supplementary material for: “Every Gene Is Everywhere but the Environment Selects”: Global Geolocalization of Gene Sharing in Environmental Samples through Network Analysis
Source: Genome Biol Evol. 2016 Apr 29;8(5):1388–400. doi: 10.1093/gbe/evw077 (PMC4898794; doi:10.1093/gbe/evw077)
Supplement: Supplementary Data [file supp_8_5_1388__index.html]

Every gene is everywhere but the environment selects: Global geo-localization of gene sharing in environmental samples through network analysis — “Every Gene Is Everywhere but the Environment Selects”: Global Geolocalization of Gene Sharing in Environmental Samples through Network Analysis — Supplementary Data 

# “Every Gene Is Everywhere but the Environment Selects”: Global Geolocalization of Gene Sharing in Environmental Samples through Network Analysis

## Supplementary Data

files

- Supplementary Data - zip file
