# Supplementary material for: “Every Gene Is Everywhere but the Environment Selects”: Global Geolocalization of Gene Sharing in Environmental Samples through Network Analysis
Source: Genome Biol Evol. 2016 Apr 29;8(5):1388–400. doi: 10.1093/gbe/evw077 (PMC4898794; doi:10.1093/gbe/evw077)
Supplement: Supplementary Data [file supp_evw077_suppl_data.zip › Fondi_et_al_SupplementaryMaterial_S1_revision2.docx]

Supplementary Material S1

*Every gene is everywhere* but *the environment selects*: Global geo-localization of gene sharing in environmental samples through network analysis

**Authors:** Fondi M^1,2,^ ^†^, Karkman A ^3,†^, Tamminen M ^4^, Bosi E ^1,2^, Virta M ^3^, Fani R ^1,2^, Alm E ^5^, McInerney JO ^6,7^*.

**Affiliations:**

^1^ Laboratory of Microbial and Molecular Evolution, Department of Biology, University of Florence, Via Madonna del Piano 6, Sesto Fiorentino, Florence, 50019, Italy.

^2^ Computational Biology Group, University of Florence, Via Madonna del Piano 6, Sesto Fiorentino, Florence, 50019, Italy.

^3^Department of Food and Environmental Sciences, University of Helsinki, P.O. Box 56, 00014 Helsinki, FINLAND

^4^ Department of Environmental Systems Science, ETH Zürich, Switzerland

^5^Department of Civil and Environmental Engineering, Massachusetts Institute of Technology, Cambridge, MA 02139, USA

^6^Department of Biology, National University of Ireland Maynooth, Maynooth, County Kildare, Ireland

^7^ Computational Evolutionary Biology, Faculty of Life Sciences, The University of Manchester, Oxford Road, Manchester M13 9PL, UK.

*Author for Correspondence: James McInerney, Computational Evolutionary Biology, Faculty of Life Sciences, The University of Manchester, Oxford Road, Manchester M13 9PL, UK.,

Email: james.mcinerney@manchester.ac.uk

^†^ Equal contributors

**Figure S1** A summary of the main features of contigs embedded in our dataset and corresponding BLAST hits. Box plots and density distribution of both retrieved contigs (A) and (B) BLAST hits within the assembled dataset. Homology searches were performed using blastn options of the BLAST tool (Altschul, et al. 1997). From this dataset, only hits longer than 500 bp and with e-value lower than 1e^-100^ were considered for further analysis.


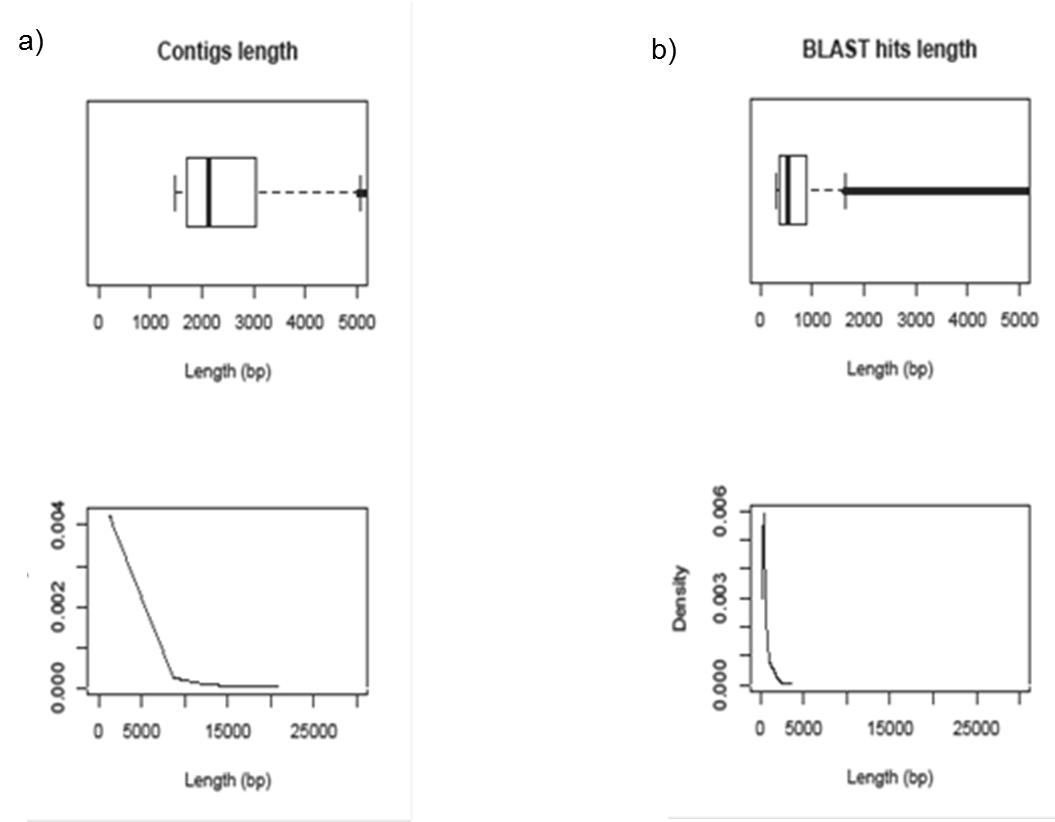


**Fig. S2. Correlation between shared hits and geographical distance.**  Scatterplot showing the absence of correlation between number of shared BLAST hits [normalized to correct for sequence content of each dataset, Y-axis) and physical distance among them expressed in Kilometres (X-axis)].

**
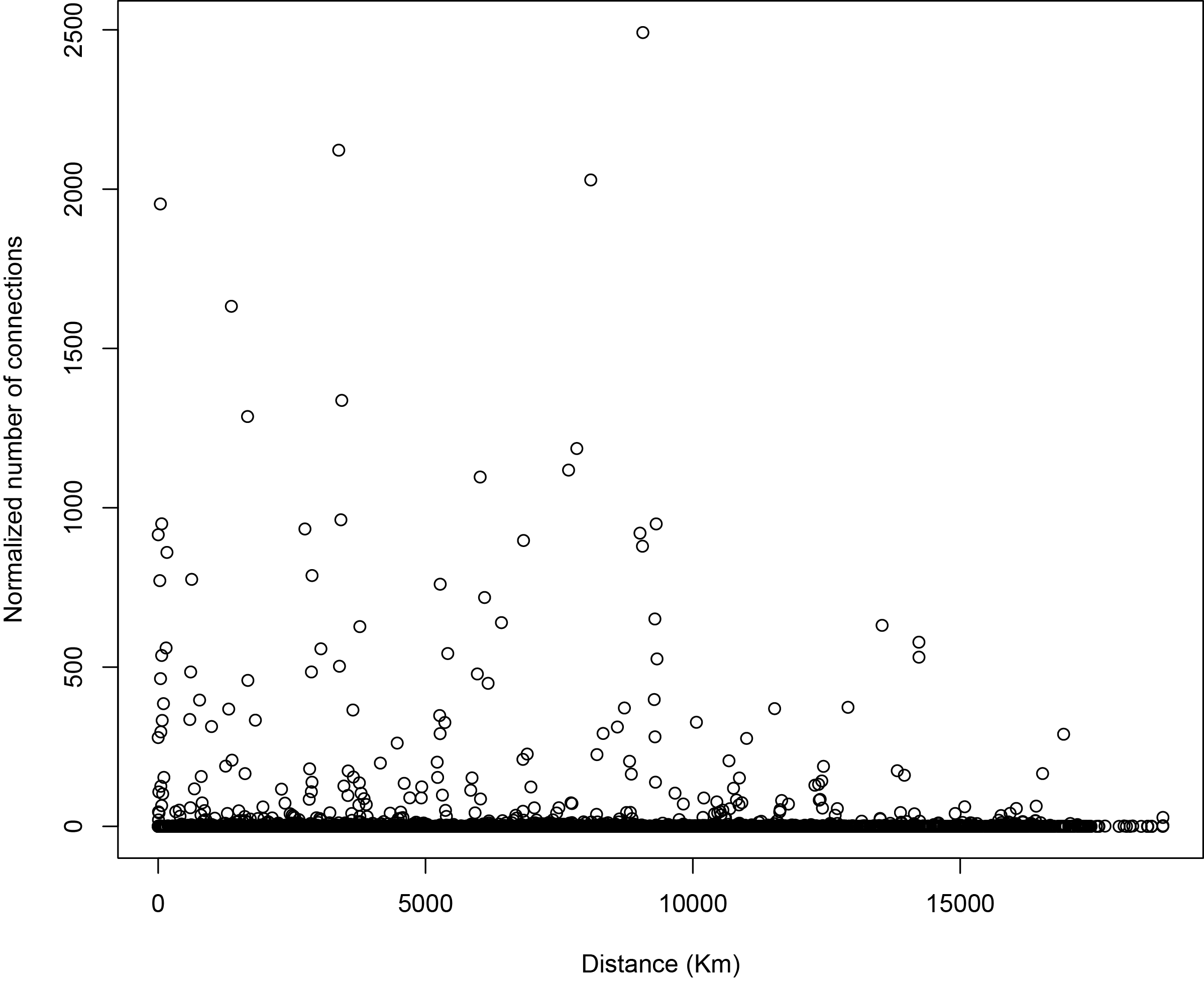
**

**Fig. S3.** Full data for Recall, Precision and Accuracy values for real and random networks at different identity thresholds (A-D) and for marker genes (E).


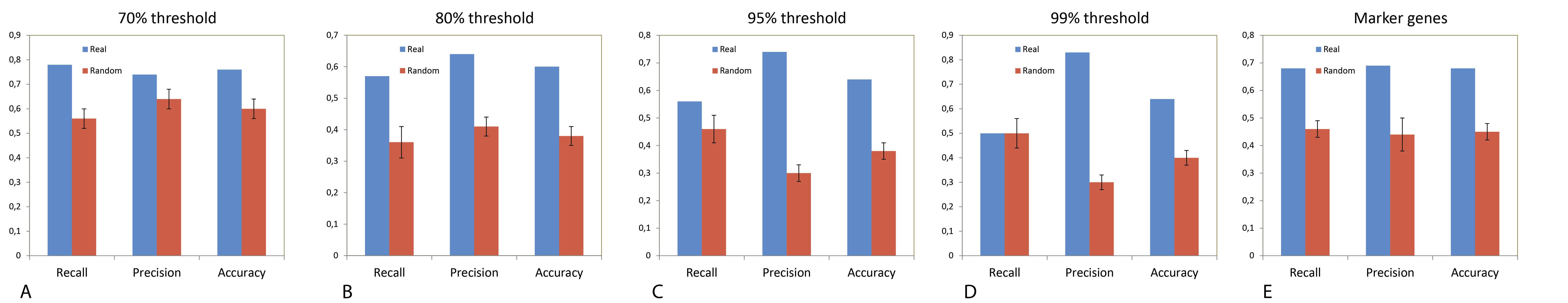


**Fig. S4.** Scatterplot showing the absence of correlation between number of shared marker genes and physical distance among them expressed in Kilometres (X-axis))..


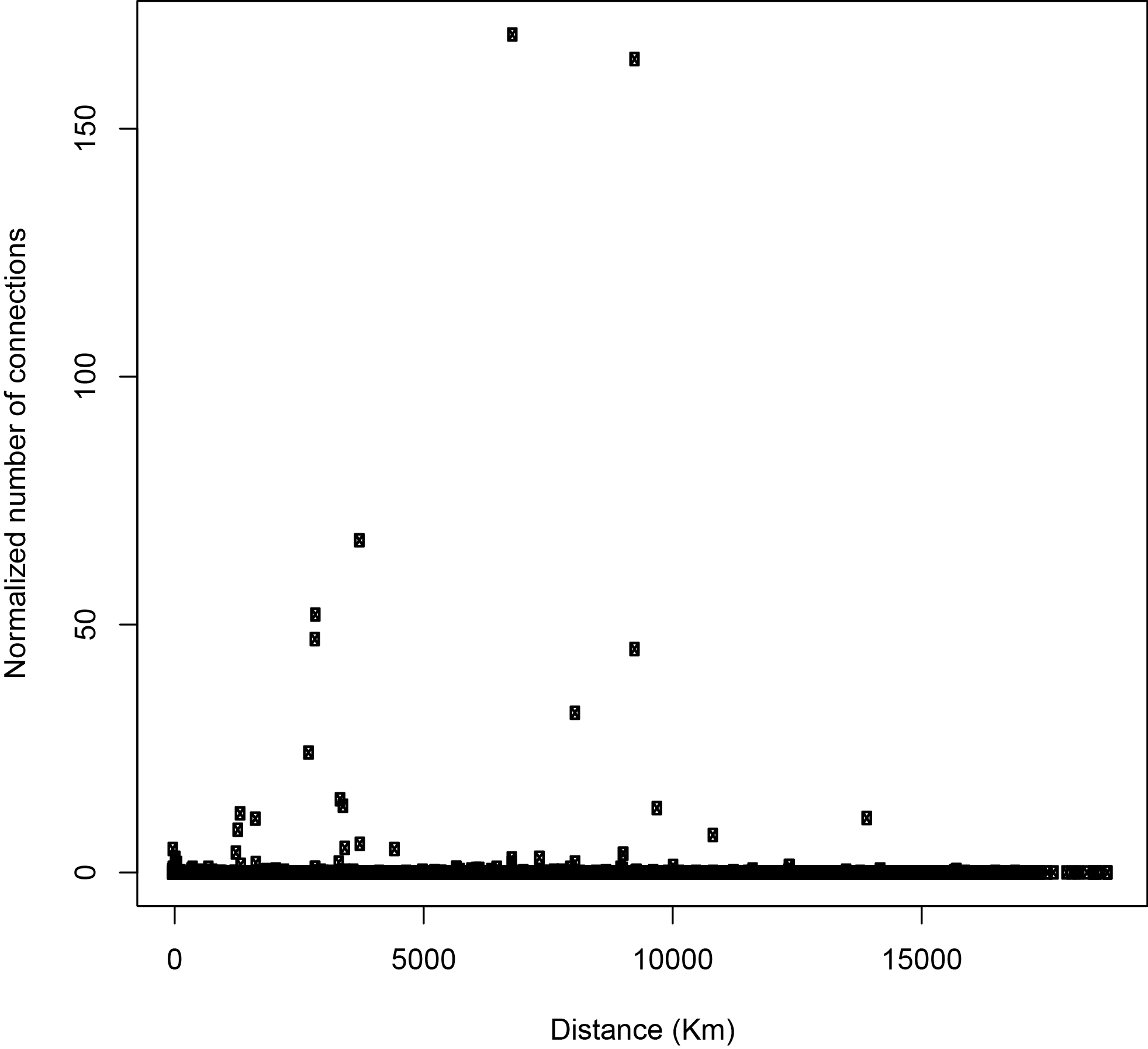


**Fig. S5.** The trend of intra-cluster cluster coefficient (red line) and the number of clusters (blue line) at the different inflation factor values tested


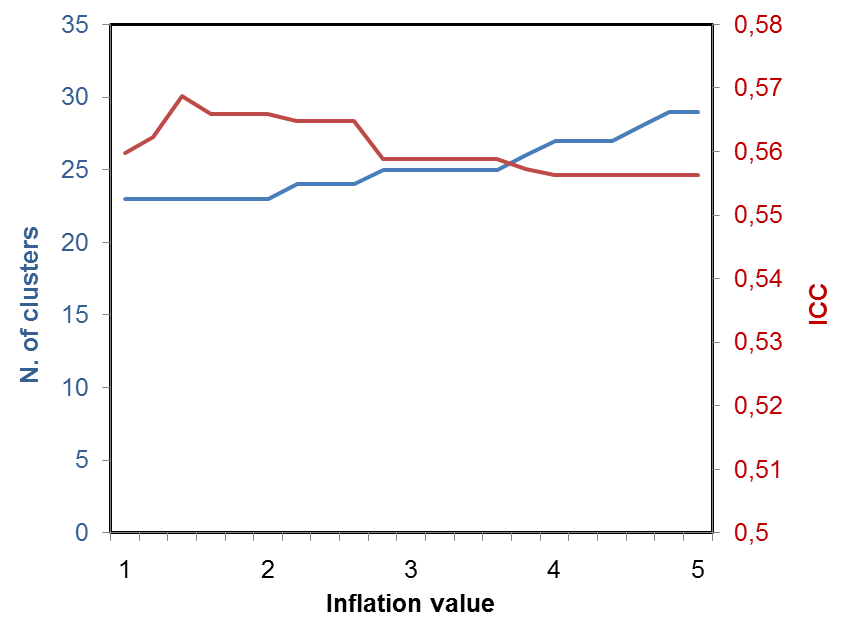


**Fig. S6.** Full data for A) relationships among number of clusters and inflation values for 70% to 99% identity networks and B) the trend of the ICCC and the number of clusters at different IF


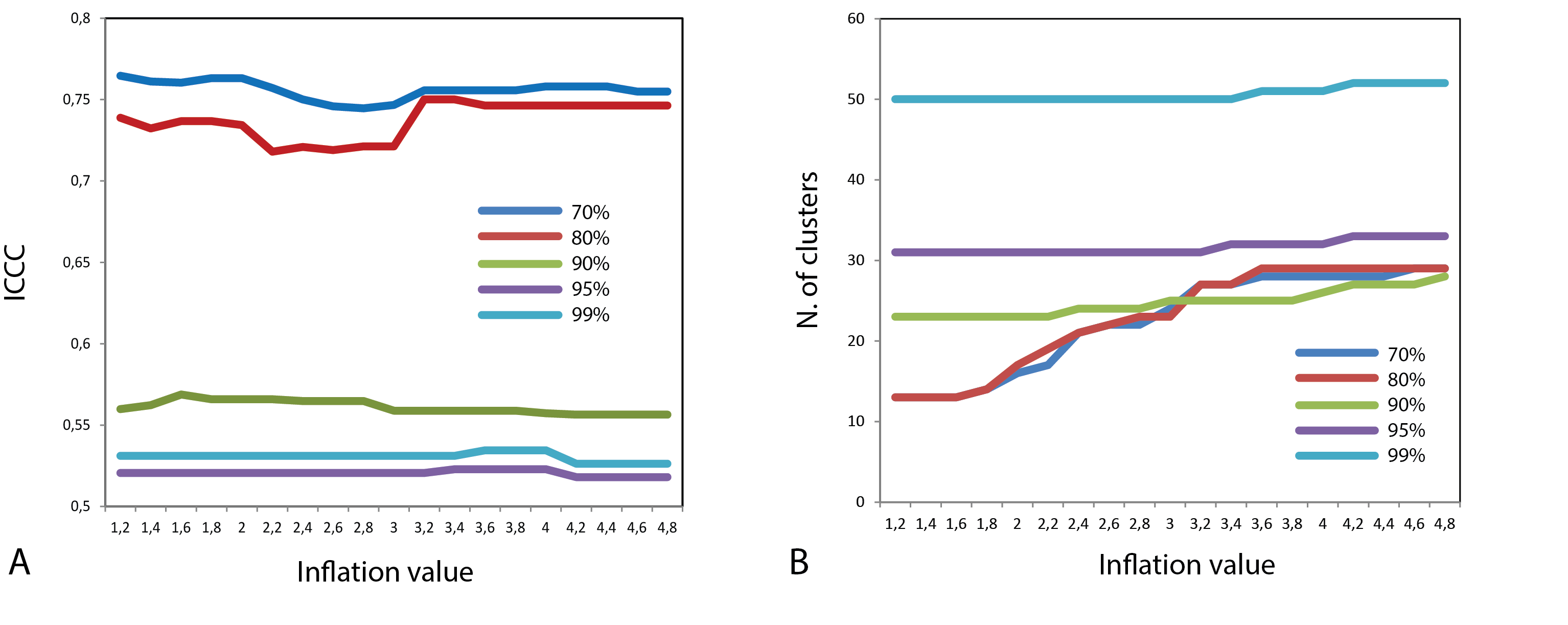


**Fig. S7.** Inter- and intra-niche evolutionary distance among sequences from the most represented samples, soil (A), sea water (B), inland water (C) and host associated (D).


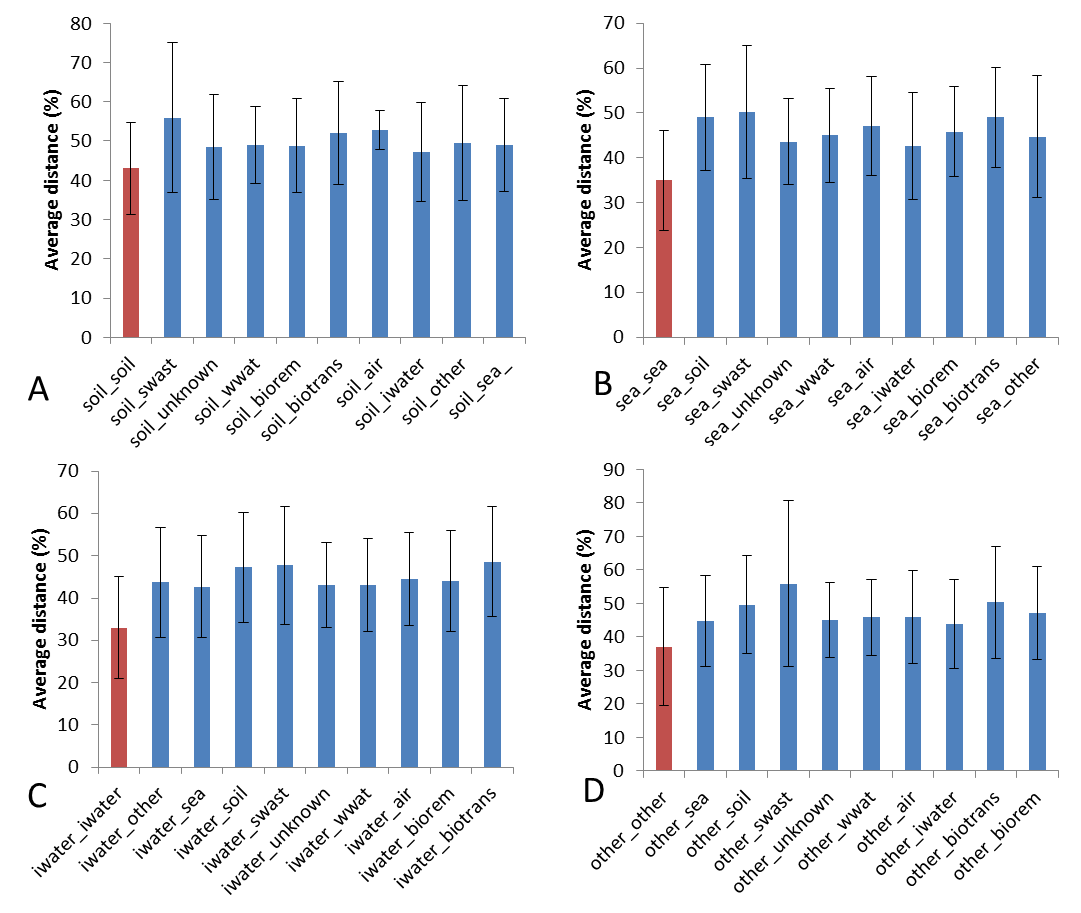


To test whether physical distance and evolutionary distance of the shared genes by the different metagenomes correlate, we calculated Pearson-product-moment correlation coefficients (PCC) for each homologous genes pair in the samples shown above. A PCC = 0.024 ( p-value = 3x10^-3^) revealed the absence of such a correlation.
